# Effects of epicatechin on cardiovascular function in middle-aged diet-induced obese rat models of metabolic syndrome

**DOI:** 10.1017/S000711452300209X

**Published:** 2024-02-28

**Authors:** Kylie Connolly, Romeo Batacan, Douglas Jackson, Andrew Stuart Fenning

**Affiliations:** 1 School of Health, Medical and Applied Sciences, Central Queensland University, Bruce Highway, Rockhampton, QLD 4701, Australia; 2 Australian Catholic University, 40 Edward St, North Sydney, NSW 2060, Australia

**Keywords:** Epicatechin, Metabolic syndrome, Diet-induced obesity, CVD, High-fat high-carbohydrate diet

## Abstract

The current study aimed to investigate the cardiovascular effects of epicatechin, a flavonoid found in green tea and cocoa, in attenuating complications associated with metabolic syndrome in diet-induced obese rats. Male Wistar-Kyoto (WKY) rats aged 16 weeks were fed either standard rat chow or given a high-fat-high-carbohydrate (HFHC) diet for 20 weeks. Epicatechin treatment (5 mg/kg/d) was administered to a subset of WKY rats commencing at week 8 of the 20 week HFHC feeding period. Body weights, food, water and energy intakes, blood pressure, heart rate and glucose tolerance were measured throughout the treatment period. Oxidative stress and inflammatory markers, lipid levels, cardiac collagen deposition, cardiac electrical function, aortic and mesenteric vessel reactivity were examined after the treatment. Twenty weeks of HFHC feeding in WKY rats resulted in the development of metabolic syndrome indicated by the presence of abdominal obesity, dyslipidaemia, glucose intolerance and increased blood pressure. Epicatechin treatment was found to enhance the oxidative stress status in HFHC groups through an increase in serum nitric oxide levels and a decrease in 8-isoprostane concentrations. Furthermore, WKY-HFHC rats displayed a decrease in IL-6 levels. The lipid profiles in HFHC groups showed improvement, with a decrease in LDL-cholesterol and TAG and an increase in HDL-cholesterol levels observed in WKY-HFHC rats. However, epicatechin was not effective in preventing weight gain, glucose intolerance or hypertension in HFHC fed rats. Overall, the results of this study suggest that epicatechin has the potential to improve the underlying mechanisms associated with metabolic syndrome in obese rats.

Research into natural compounds that can help prevent or treat CVD is increasingly popular in the scientific community. Polyphenolic flavonoids, which are found in many foods, have been shown to have beneficial effects on cardiovascular function^(1–3)^. One such flavonoid is epicatechin, which is abundant in green tea, grapes and cocoa. Epicatechin has multiple properties that can help reduce cardiovascular risk, including its anti-inflammatory, antioxidant and antihypertensive abilities^(2,4–6)^. However, further research is necessary to determine the exact mechanism of action of dietary flavanols like epicatechin, given their complex activity and multiple cellular and enzymatic targets.

Studies on epicatechin’s therapeutic effects are increasing, with various doses being tested in animal models. For example, dietary administration of epicatechin at a high dose (4 g/kg/diet equivalent to an average consumption of 304 mg/kg/d) for 4 d prevented hypertension and oxidative stress in nitric oxide deficient rats treated with L-N^G^-Nitro arginine methyl ester^(7)^. In contrast, a lower dose of epicatechin (10 mg/kg/d for 4 weeks) did not prevent hypertension but reduced cardiac hypertrophy, decreased vascular NADPH oxidase activity and increased endothelial nitric oxide synthase phosphorylation in rats treated with L-N^G^-Nitro arginine methyl ester^(8)^. Smaller doses of epicatechin also provide protective effects in animal studies, such as preventing weight gain, decreasing blood glucose and TAG, and improving mitochondrial function in obese rats given 1 mg/kg/d for 2 weeks^(9)^.

In addition to its therapeutic effects, studies on the absorption, distribution and secretion of epicatechin in rats have revealed interesting findings which have helped to establish its oral bioavailability^(10)^. Plasma concentrations of epicatechin peak rapidly 1 hour after oral ingestion before gradually declining, but they again begin to increase 6 hours after ingestion^(10)^. This suggests that the fermented metabolites of epicatechin may be absorbed later in the digestion process, unlike many other flavonoids. Furthermore, epicatechin metabolites may be undergoing reabsorption after biliary excreted conjugation, and these metabolites may also have cardioprotective activity^(10)^.

While there are reports in the literature that detail the effects of diet-induced obesity in metabolic syndrome, these works are largely focused on insulin resistance, lipid profiles, inflammation and adipose tissue distribution^(11,12)^. There are limited studies available reporting the effects of diet-induced obesity on vascular reactivity, cardiac electrophysiology and cardiac remodelling induced by fibrosis in the obese rat model. This highlights the substantial gap in the current knowledge base of obesity-related CVD.

Despite the increasing number of studies investigating the potential of epicatechin in treating cardiovascular and obesity-related disorders, much remains to be understood about epicatechin’s therapeutic effects in both chronic and mechanistic contexts. Therefore, this study aimed to assess the ability of 12 weeks of epicatechin treatment (5 mg/kg/d) to prevent cardiovascular dysfunction in obese middle-aged rat models of metabolic syndrome.

## Materials and methods

### Study design and animals

The Animal Ethics Research Committee (AEC approval #A12/04–281) at CQUniversity approved all animal handling and experimental treatments conducted in this project. The ARRIVE guidelines author checklist was used for this animal study to ensure comprehensive and transparent reporting of experimental methods, results and data analysis^(13)^. Male Wistar Kyoto (WKY) rats, aged between 4 and 10 weeks, were obtained from the Animal Resource Centre in Perth, Western Australia. The rats were housed in groups of three in standard plastic cages, under a 12-hour light/dark cycle, at a constant temperature of 22°C ± 2°C in CQUniversity’s animal house. Throughout the experiment, the rats had unlimited access to water and standard rat chow pellets until they reached 16 weeks of age. The rats (mean body weight 314 ± 5·0) were then randomly divided into four treatment groups: WKY, WKY + Epicatechin, WKY-HFHC and WKY-HFHC + Epicatechin (*n* 11 per group).

The rats in the HFHC groups were fed a high-fat, high-carbohydrate diet (23·9 % fat, 51·5 % carbohydrate; with fructose 25 % in drinking water) for 20 weeks, while the control rats were fed standard chow. The HFHC diet composition was based on the diets used in previous studies of obesity and metabolic syndrome^(14,15)^ and was prepared on-site at CQUniversity. The composition of animal diets is listed in Table 1. The WKY-HFHC received fructose (25 % solution) in their drinking water throughout the HFHC feeding period, and both HFHC food and fructose solution were refreshed daily. All rats were monitored daily for sufficient food and fresh water, clean cages and good health throughout the experiment.

**Table 1.** Composition of animal diets

Ingredients	Standard chow	HFHC Feed	Fructose solution
Standard rat chow g/kg	1000		
Powdered rat feed g/kg	–	155	–
HMW salt mixture g/kg	–	25	–
Beef tallow g/kg	–	200	–
Condensed milk g/kg	–	395	–
Fructose g/kg	–	175	250
Water ml/kg	–	50	1000
Energy kJ/g	12·2	17·9	16·8
Macronutrients			
Total carbohydrate %	42	52	100
Total fat %	5	24	–
Total protein %	25	6	–
Total fibre %	6	1	–

Standard rat chow/powdered rat feed ((Riverina Stockfeeds, South Brisbane, Australia); HMW salt mixture (Kemix Pty Ltd Victoria, Australia); Beef tallow/Red band beef shortening (Campbells Wholesale Rockhampton); Condense milk (Woolworths Rockhampton); Fructose (Melbourne Food Depot).

After 8 weeks of HFHC feeding, rats in the epicatechin (5 mg/kg/d) groups received 12 weeks of treatment via oral gavage while continuing the HFHC diet. The dose and duration of epicatechin treatment were selected to deliver cardio-beneficial effects that would occur independently of blood pressure reduction in an obese hypertensive rat model.

### Drug and compounds

Epicatechin, noradrenaline, acetylcholine and sodium nitroprusside were sourced from the Sigma Chemical Company. MilliQ water was used to prepare serial dilutions of noradrenaline, acetylcholine and sodium nitroprusside. Epicatechin was also prepared using MilliQ water to achieve a final concentration of 5 mg/ml.

### Body weight, energy, food and water/fructose intake

Throughout the treatment period, the body weights, 24-h food and water/fructose intakes of all groups were recorded every week. The total energy intakes were determined by multiplying the kJ value per gram of food/fructose, as provided in Table 1.

### Systolic blood pressure and heart rate

Systolic blood pressure and heart rate were measured using tail-cuff plethysmography at treatment weeks 0, 4, 8, 12, 16 and 20. To induce a light anaesthetic state, each rat was given an intraperitoneal injection of 0·1 ml Zoletil® (Tiletamine 15 mg/kg and Zolazepam 15 mg/kg). A pulse transducer (MLT1010) connected to a Capto SP844 pressure transducer (MLT844/D) and a Powerlab data unit (AD Instruments) was used to record readings from the base of the rat’s tail. Three consecutive recordings of systolic blood pressure and heart rate were taken and averaged for each rat.

### Oral glucose tolerance test

At treatment weeks 0, 8 and 20, oral glucose tolerance tests were performed. Following an overnight fast, a 40 % glucose solution (2 g/kg/bwt) was orally administered to each rat. Blood glucose levels were measured from unanesthetised rats by pricking the tail tip using a disposable lancet and applying 2 ul of blood to an activated glucometer test strip. Blood samples were collected at 0, 30, 60, 90 and 120 min after glucose intake and measured by a Redi-cote Advocate standard glucometer to evaluate progressive glucose intolerance. The oral glucose tolerance test was assessed by determining the area under the curve using the trapezoidal rule.

### Biochemical assay

At the end of the treatment period, rats were euthanised through intraperitoneal injection of sodium pentobarbitone, which was prepared at a concentration of 187·5 mg/ml. A 5 ml blood sample was drawn from the abdominal vena cava and left to clot in serum separator tubes, followed by centrifugation for 10 min at 4000 rpm. The kidneys, spleen, liver, abdominal fat, epididymal fat and left and right ventricles were removed and weighed to assess organ hypertrophy. Serum samples, as well as sections of the left ventricle, were frozen in liquid nitrogen and stored at −80°C for further use.

The R&D Systems ELISA duo set (catalogue number: DY506) was used to analyse serum IL-6 concentrations. The Cayman Chemicals Express EIA kit (catalogue number 516 360) was used to assess serum concentrations of 8-isoprostane. Abcam HDL and LDL/VLDL Cholesterol Assay Kits (catalogue number ab65390) were used to measure HDL and LDL serum levels, respectively. The Abcam Triglyceride Quantification Kit (catalogue number ab65336) was used to determine serum TAG concentrations. The amount of procollagen type I in serum was measured using My BioSource Rat PICP (Procollagen Type I Carboxyterminal Propeptide) ELISA Kit (catalogue number MBS2502976). Standards and samples were prepared in accordance with the manufacturer’s instructions. The Sorte and Basak^(16)^ modified copper–cadmium reduction method was employed to quantify total nitric oxide concentrations.

### Vascular function

To evaluate the vascular function, 4 mm segments of thoracic aorta were placed in 25 ml organ baths filled with gassed (5 % CO_2_ and 95 % O_2_) Tyrode solution at 37°C with a pre-set resting tension of 10 mN. After equilibration for 30 min, cumulative concentrations of noradrenaline (10^−9^ M to 10^−4^ M) were added to assess vascular contraction. After a washout period and an additional 30 min of equilibration, tissues were pre-contracted with a single dose of noradrenaline (10^−6^ M) and allowed to reach a plateau. To evaluate endothelium-dependent and independent relaxation, acetylcholine or sodium nitroprusside (10^−9^ M to 10^−4^ M) was cumulatively added to the baths. Tissue responses were measured with transducers (Grass FT03) and recorded using Chart software.

For mesenteric arteries, 2 mm segments were mounted in 10 ml chambers of the Mulvaney myograph system (Model 610 M, Version 2·2, Danish Myo Technology) filled with gassed (5 % CO_2_ and 95 % O_2_) Tyrode solution at 37°C. After a normalisation procedure following the manufacturer’s guidelines, tissues were allowed to equilibrate for 30 min. Cumulative concentrations of noradrenaline (10^−9^ M to 10^−4^ M) were added to the baths to assess vascular contraction, followed by a washout period and an additional 30 min of equilibration. Tissues were then pre-contracted with noradrenaline (10^−5^ M) and allowed to reach a plateau before acetylcholine or sodium nitroprusside (10^−9^ M to 10^−4^ M) were cumulatively added to the baths to assess endothelium-dependent and independent relaxation. Tissue responses were recorded using an Apple intel iMac computer connected to a PowerLab 8 SP recorder running Chart software.

### Cardiac function

The left ventricular papillary muscles were dissected from the heart and placed in cold, gassed (95 %-O_2_ and 5 %-CO_2_) Tyrode solution. A stainless-steel hook was inserted into the superior end of the muscle, which was then positioned in a 1 ml experimental chamber that was perfused with warm Tyrode solution at a flow rate of 3 ml/min. The inferior end of the muscle was pinned to a rubber base, and a modified sensor element (SensoNor AE801) attached to an amplifier (World Precision Instruments, TBM-4) was used to measure the muscle’s contractions. The muscle was gradually extended over one minute to obtain a maximum preload of 5–10 mN. Field stimulation was initiated using a Grass SD-9 stimulator to produce contractions at a frequency of 1 Hz, pulse width of 0·5 msec and stimulus strength 20 % above the threshold. A potassium chloride glass electrode (World Precision Instruments) with a tip resistance of 5–15 mΩ was used to impale the muscle. The electrical activity was recorded using a Cyto 721 electrometer (World Precision Instruments) and an iMac G5 computer incorporating an Analogue Digital Converter (PowerLab 4/25) for a period of 20 min to obtain control readings. Data were analysed using PowerLab Chart 5·5 software (AD Instruments), including measurements of action potential duration at 20 %, 50 % and 90 % of repolarisation (APD20, APD50 and APD90, respectively), resting membrane potential, action potential amplitude and force of contraction.

### Statistical analysis

A power analysis was conducted using G-power 3·1 software with an *α* level of 0·05, a power *β* level of 0·95 and an effect size of 0·33 to determine sample sizes needed to detect significant differences in this study. These were based on the functional outcome measures for the cardio-electrophysiological and blood vessel experiments. Normality was evaluated by Kolmogorov–Smirnov and Shapiro–Wilk tests. Time and group interactions were tested using generalised estimating equations with an unstructured correlation matrix for repeated longitudinal measures. GraphPad Prism software was employed to analyse biochemical and cardiovascular function data using two-way ANOVA, with subsequent Bonferroni *post hoc* tests and student’s *t* tests where applicable. Concentration–response curves were fitted using GraphPad Prism (v4) to determine pEC50 values. Results were considered significant at *P* < 0·05 and are presented as mean ± sem.

## Results

### Effect of epicatechin on body weight, energy, food and water/fructose intake in Wistar-Kyoto and Wistar-Kyoto-high-fat-high-carbohydrate rats

After 12 weeks of intervention, there were no significant differences in body weights between the groups, and no significant interactions between group and time were observed. However, an increase in body weight was noted for all groups starting from week 6 onwards (Table 2). While energy intake remained consistent across different time points and no significant interaction between group and time was detected, rats on the HFHC diet showed significantly increased energy consumption compared with standard chow fed rats (Table 3). The WKY-HFHC and the WKY-HFHC + E groups had the same amount of weight gain across the duration of the experiment (Table 4).

**Table 2.** GEE analysis of body weights of treatment and control groups

Weeks				WKY	WKY + E			WKY-HFHC			WKY-HFHC + E		
	*β*	95 % CI	*P*-value	*β* (95 % CI)	*β*	95 % CI	*P*-value	*β*	95 % CI	*P*-value	*β*	95 % CI	*P*-value
				Ref*	9·16	–13·67, 31·98	0·43	5·79	–12·62, 24·20	0·54	16·70	–2·53, 35·93	0·09
1	Ref†			Ref‡	Ref‡			Ref‡			Ref‡		
2	11·40	–10·33, 33·13	0·30	Ref‡	1·24	–30·53, 33·00	0·94	–14·31	–40·98, 12·36	0·29	–23·94	–50·84, 2·95	0·08
3	14·70	–6·37, 35·77	0·17	Ref‡	3·76	–28·10, 35·61	0·82	–11·06	–38·51, 16·39	0·43	–11·06	–37·75, 15·62	0·42
4	19·80	–1·26, 40·86	0·06	Ref‡	2·74	–29·19, 34·68	0·87	–8·71	–38·38, 20·96	0·56	–12·34	–41·02, 16·33	0·40
5	20·20	–0·62, 41·02	0·06	Ref‡	10·80	–21·43, 43·03	0·51	–12·02	–42·54, 18·50	0·44	–11·66	–39·54, 16·23	0·41
6	24·70	3·38, 46·02	0·02	Ref‡	7·84	–25·04, 40·73	0·64	–9·79	–41·25, 21·67	0·54	–8·24	–37·03, 20·54	0·58
7	32·40	10·20, 54·60	0·004	Ref‡	2·96	–30·60, 36·53	0·86	–10·76	–43·30, 21·77	0·52	–5·40	–35·90, 25·10	0·73
8	36·40	14·46, 58·34	0·001	Ref‡	2·69	–30·78, 36·16	0·88	–9·76	–42·59, 23·06	0·56	–5·40	–36·55, 25·75	0·73
9	35·80	14·40, 57·20	0·001	Ref‡	2·84	–29·95, 35·62	0·86	–7·07	–39·69, 25·55	0·67	–0·98	–33·04, 31·08	0·95
10	40·70	19·34, 62·06	< 0·001	Ref‡	7·12	–26·36, 40·59	0·68	–9·43	–42·25, 23·40	0·57	–1·70	–33·52, 30·12	0·92
11	44·50	23·34, 65·66	< 0·001	Ref‡	4·50	–28·92, 37·92	0·79	–6·59	–39·92, 26·74	0·70	–2·68	–34·08, 28·72	0·87
12	43·50	22·04, 64·98	< 0·001	Ref‡	4·86	–27·91, 37·63	0·77	–3·32	–37·69, 31·05	0·85	–0·86	–33·80, 32·08	0·96
13	50·40	29·08, 71·72	< 0·001	Ref‡	–5·13	–38·11, 27·85	0·76	–9·22	–46·35, 27·92	0·63	–13·22	–45·77, 19·34	0·43
14	49·50	28·60, 70·40	< 0·001	Ref‡	3·14	–29·56, 35·83	0·85	–10·86	–46·06, 24·33	0·54	–7·96	–41·45, 25·54	0·64
15	50·60	28·77, 72·43	< 0·001	Ref‡	4·86	–28·03, 37·74	0·77	–9·69	–45·97, 26·59	0·60	–5·24	–40·90, 30·42	0·77
16	52·40	30·68, 74·12	< 0·001	Ref‡	7·42	–25·24, 40·08	0·66	4·06	–27·35, 35·46	0·80	11·24	–20·91, 43·38	0·49
17	47·30	26·50, 68·10	< 0·001	Ref‡	8·43	–23·96, 40·81	0·61	18·34	–9·48, 46·15	0·20	22·61	–6·62, 51·84	0·13
18	53·50	32·43, 74·57	< 0·001	Ref‡	2·59	–29·89, 35·08	0·88	20·14	–7·59, 47·86	0·16	23·59	–5·41, 52·60	0·11
19	58·20	36·38, 80·02	< 0·001	Ref‡	0·98	–31·78, 33·74	0·95	20·26	–7·66, 48·16	0·16	22·71	–6·97, 52·39	0·13
20	62·80	41·01, 84·59	< 0·001	Ref‡	3·11	–29·78, 36·00	0·85	26·66	–0·95, 54·26	0·06	28·38	–1·56, 58·32	0·06

Results were obtained from generalised estimating equations (GEE) method. Robust estimator with an unstructured correlation matrix was used. *β*, regression coefficient; 95 % CI, 95 % Wald confidence interval; Ref, reference group.

* Reference group for group effect.

† Reference group for time effect.

‡ Reference group for group × time interaction effect.

**Table 3.** GEE analysis of energy intake of treatment and control groups

Variables	*β*	95 % CI	*P*-value
WKY	Ref*		
WKY + E	12·92	–8·61, 34·44	0·24
WKY-HFHC	78·19	45·23, 111·15	< 0·001
WKY-HFHC + E	77·52	50·20, 104·85	< 0·001
Weeks	–0·98	–2·32, 0·35	0·15
WKY × Weeks	Ref†		
WKY + E × Weeks	–0·10	–2·66, 0·67	0·24
WKY-HFHC × Weeks	0·92	–1·78, 3·63	0·50
WKY-HFHC + E × Weeks	0·82	–1·48, 3·13	0·15

Results were obtained from generalised estimating equations (GEE) method. Robust estimator with an unstructured correlation matrix was used. *β*, regression coefficient; 95 % CI, 95 % Wald confidence interval; Ref, reference group.

* Reference group for group effect.

† Reference group for group × time interaction effect.

**Table 4.** Body weights, energy, food and water/fructose intakes of treatment and control groups

Parameters	WKY		WKY + E		WKY-HFHC		WKY-HFHC + E	
	Mean	sem	Mean	sem	Mean	sem	Mean	sem
Initial body weight (g) (at 0 weeks)	302·91	7·68	311·45	8·85	308·09	5·11	319·0	5·92
Body weight (g) (at 8 weeks)	338·10	7·80	350·09	9·92	336·82	12·14	350·00	10·24
Final body weight (g) (at 20 weeks)	365·10	8·11	377·36	9·77	397·55	7·49*	410·18	9·26*
Body weight increase from 8 weeks (%)	8·01	0·56	7·78	0·52	18·03	2·31*	17·19	1·95*
Body weight increase from 14 weeks (%)	3·48	0·45	2·88	0·42	15·07	2·87*	13·44	2·74*
Food intake from 8 weeks (g/d)	20·36	2·26	20·04	1·19	12·08	1·28*	11·66	1·06*
Water or fructose intake from 8 weeks (ml/d)	32·35	3·18	31·68	1·72	22·31	2·54	23·04	2·15
Energy intake from 8 weeks (kJ/d)	225·81	25·10	244·46	14·47	320·56	34·96*	315·90	29·17*

Food, water/fructose and energy intakes are averaged values for the duration of the study. E, epicatechin; HFHC, high-fat, high-carbohydrate. Data expressed as mean ± sem; *n* 8–11 for all groups.

* *P* < 0·05 *v*. WKY (two-way ANOVA with Bonferroni’s multiple comparisons tests).

Epicatechin treatment had no significant effect on food or fructose consumption or energy intake in either HFHC or standard chow fed rats (Table 4). Twelve weeks of epicatechin treatment had no effect in reducing weight gain in WKY or HFHC fed rats (Table 4).

### Effect of epicatechin on organ weights in Wistar-Kyoto and Wistar-Kyoto-high-fat-high-carbohydrate rats

Epicatechin treatment was unable to attenuate abdominal and epididymal weight gain (fat mass) in WKY-HFHC rats (Table 5). When normalised for body weight, epicatechin treatment reduced liver weight in WKY rats compared with control rats. This trend was also observed in epicatechin-treated WKY-HFHC rats; however, the decrease was not considered significant (Table 5). Differences in left ventricle weights remained non-significant in both raw weight (g) and when normalised for body weight (mg/g bwt) amongst all groups.

**Table 5.** Organ weights of treatment and control groups

Parameters	WKY		WKY + E		WKY-HFHC		WKY-HFHC + E	
	Mean	sem	Mean	sem	Mean	sem	Mean	sem
Abdominal fat (mg/g bwt)	15·41	1·39	14·73	1·02	30·39	1·18*	32·17	2·26*
Epididymal fat (mg/g bwt)	12·20	0·84	11·70	0·50	17·56	0·89*	20·31	1·06*,†
Left ventricle (mg/g bwt)	3·34	0·53	2·60	0·13	2·67	0·14	2·50	0·16
Left ventricle (g)	1·33	0·19	0·98	0·10	1·06	0·09	1·03	0·13
Right ventricle (mg/g bwt)	1·01	0·17	0·64	0·09*	0·61	0·06*	0·67	0·09*
Kidneys (mg/g bwt)	6·44	0·08	6·46	0·09	6·22	0·12	5·96	0·10
Spleen (mg/g bwt)	1·61	0·09	1·56	0·06	1·44	0·08	1·44	0·05
Liver (mg/g bwt)	27·94	0·80	26·02	0·42*	28·21	0·74	27·38	0·59

E, epicatechin; HFHC, high-fat, high-carbohydrate. Data expressed as mean ± sem; *n* 8–11 for all groups.

* *P* < 0·05 *v*. WKY.

† *P* < 0·05 *v*. WKY-HFHC (two-way ANOVA with Bonferroni’s multiple comparisons tests).

### Effect of epicatechin on glucose tolerance in Wistar-Kyoto and Wistar-Kyoto-high-fat-high-carbohydrate rats

Although no significant difference was found in the oral glucose tolerance test results between groups and across different time points, significant interactions were observed between group and time (Table 6). Glucose tolerance was impaired in HFHC fed rats after 16 weeks of HFHC feeding as indicated by a significant increase in AUC values during weeks 16 and 20 (Table 6).

**Table 6.** GEE analysis of oral glucose tolerance (AUC) of treatment and control groups

Weeks				WKY	WKY + E			WKY-HFHC			WKY-HFHC + E		
	*β*	95 % CI	*P*-value	*β* (95 % CI)	*β*	95 % CI	*P*-value	*β*	95 % CI	*P*-value	*β*	95 % CI	*P*-value
				Ref*	13·30	–31·50, 58·08	0·56	–21·10	–62·97, 20·77	0·32	–17·13	–61·38, 27·12	0·45
0	Ref†			Ref‡	Ref‡			Ref‡			Ref‡		
4	21·65	–10·11, 53·41	0·18	Ref‡	–31·77	–80·77, 17·23	0·20	104·80	23·64, 185·96	0·01	47·39	–9·10, 103·88	0·10
8	104·49	5·95, 203·02	0·04	Ref‡	–140·14	–242·24, −38·04	0·01	66·50	–71·61, 204·62	0·34	–24·20	–130·39, 81·99	0·66
12	63·65	–14·83, 142·12	0·11	Ref‡	8·48	–107·87, 124·83	0·87	43·36	–76·87, 163·58	0·48	34·77	–84·34, 153·87	0·57
16	72·08	–21·22, 165·38	0·13	Ref‡	–20·35	–122·24, 81·53	0·70	133·48	17·29, 249·67	0·02	100·37	–3·10, 203·84	0·06
20	101·40	–10·08, 212·88	0·08	Ref‡	15·42	–99·32, 130·15	0·79	149·87	5·16, 294·58	0·04	158·94	22·73, 295·14	0·02

Results were obtained from generalized estimating equations (GEE) method. Robust estimator with an unstructured correlation matrix was used. *β*, regression coefficient; 95 % CI, 95 % Wald confidence interval; Ref, reference group.

* Reference group for group effect.

† Reference group for time effect.

‡ Reference group for group × time interaction effect.

### Effect of epicatechin on systolic blood pressure and heart rate in Wistar-Kyoto and Wistar-Kyoto-high-fat-high-carbohydrate rats

There was no significant difference found in blood pressure between groups and across different time points, but significant interactions were observed between group and time (Table 7). WKY-HFHC and WKY-HFHC + E groups developed significantly increased systolic blood pressure during the last 8 weeks of HFHC feeding compared with WKY rats (Table 7). Epicatechin was not able to prevent the increase in systolic blood pressure induced by HFHC feeding in WKY rats (Table 7). There was no significant difference in heart rate readings between groups (χ^2^ = 59·64; *P* = 0·57), across different time points (χ^2^ = 20·78; *P* = 0·84) and no group and time interaction (χ^2^ = 12·55; *P* = 0·64) was observed.

**Table 7.** GEE analysis of blood pressure of treatment and control groups

Weeks				WKY	WKY + E			WKY-HFHC			WKY-HFHC + E		
	*β*	95 % CI	*P*-value	*β* (95 % CI)	*β*	95 % CI	*P*-value	*β*	95 % CI	*P*-value	*β*	95 % CI	*P*-value
				Ref*	–1·53	–13·36, 10·30	0·80	–1·14	–10·18, 7·89	0·80	–0·96	–10·02, 8·10	0·83
0	Ref†			Ref‡	Ref‡			Ref‡			Ref‡		
4	2·42	–5·12, 9·96	0·53	Ref‡	–1·73	–11·42, 7·95	0·73	6·37	–5·18, 17·91	0·28	4·60	–7·27, 16·47	0·45
8	1·70	–6·11, 9·50	0·67	Ref‡	–1·16	–13·20, 10·87	0·85	16·58	6·71, 26·45	< 0·001	6·96	–3·77, 17·69	0·20
12	4·38	–3·93, 12·68	0·30	Ref ‡	4·48	–8·52, 17·49	0·50	24·79	12·44, 37·14	< 0·001	17·17	4·48, 29·87	0·01
16	14·08	2·11, 26·06	0·02	Ref‡	0·44	–15·23, 16·11	0·96	24·18	10·84, 37·52	< 0·001	16·53	1·48, 31·59	0·03
20	8·86	–1·82, 19·55	0·10	Ref‡	7·93	–7·32, 23·17	0·31	31·51	19·11, 43·91	< 0·001	18·09	3·41, 32·77	0·02

Results were obtained from generalised estimating equations (GEE) method. Model-based estimator with an unstructured correlation matrix was used. *β*, coefficient; DSS, dextran sodium sulphate; LGH, longish glucomannan hydrolysates; Ref, reference group.

* Reference group for group effect

† Reference group for time effect.

‡ Reference group for group × time interaction effect.

### Effect of epicatechin on serum markers of oxidative stress, inflammation, lipid profile and procollagen type I carboxyterminal propeptide in Wistar-Kyoto and Wistar-Kyoto-high-fat-high-carbohydrate rats

Serum nitric oxide was significantly increased in epicatechin-treated HFHC rats compared with control rats (Table 8). Serum concentrations of TAG were significantly decreased in epicatechin treated HFHC rats compared with control rats (Table 8). Epicatechin significantly reduced serum levels of LDL in WKY-HFHC rats compared with untreated HFHC rats (Table 8). Epicatechin significantly reduced serum levels of IL-6 and 8-isoprostane in WKY-HFHC rats; however, this reduction was not observed in standard chow fed rats treated with epicatechin (Table 8). HDL were significantly increased in WKY-HFHC + E and WKY + E rats compared with control rats (Table 8). Treatment with epicatechin reduced serum PICP levels in all groups; however, the reductions did not reach significance (Table 8).

**Table 8.** Serum markers of oxidative stress, inflammation, lipid profile and procollagen type I carboxyterminal propeptide (PICP) of treatment and control groups

Parameters	WKY		WKY + E		WKY-HFHC		WKY-HFHC + E	
	Mean	sem	Mean	sem	Mean	sem	Mean	sem
Nitric oxide (µM)	27·74	1·75	27·47	1·63	28·90	1·77	44·14	3·40*,†
Interleukin-6 (pg/ml)	57·94	2·24	58·28	1·62	51·42	1·21*	20·31	1·09*,†
8-isoprostane (pg/ml)	119·67	17·49	113·03	12·66	127·34	16·78	72·56	7·91*,†
Low-density lipoprotein (mg/dl)	54·44	3·50	53·23	3·76	116·11	8·01*	79·57	7·31*,†
High-density lipoprotein (mg/dl)	46·31	5·06	63·31	5·74*	64·68	7·90*	82·76	6·92*,†
LDL:HDL(ratio)	1·18	0·20	0·84	0·10	1·79	0·37	0·96	0·13
TAG (mg/dl)	181·30	21·04	173·39	38·38	1114·11	78·03*	475·28	75·69*,†
Total cholesterol(mg/dl)	137·01	4·26	151·21	17·18	390·19	36·41*	261·31	33·02*
Procollagen type 1(mU)	0·741	0·026	0·690	0·027	0·877	0·037*	0·809	0·033

E, epicatechin; HFHC, high-fat, high-carbohydrate. Data expressed as mean ± sem; *n* 8–11 for all groups.

* *P* < 0·05 *v*. WKY.

† *P* < 0·05 *v*. WKY-HFHC (two-way ANOVA with Bonferroni’s multiple comparisons tests).

### Effect of epicatechin on vascular function in thoracic aorta of Wistar-Kyoto and Wistar-Kyoto-high-fat-high-carbohydrate rats

**Fig. 1.**
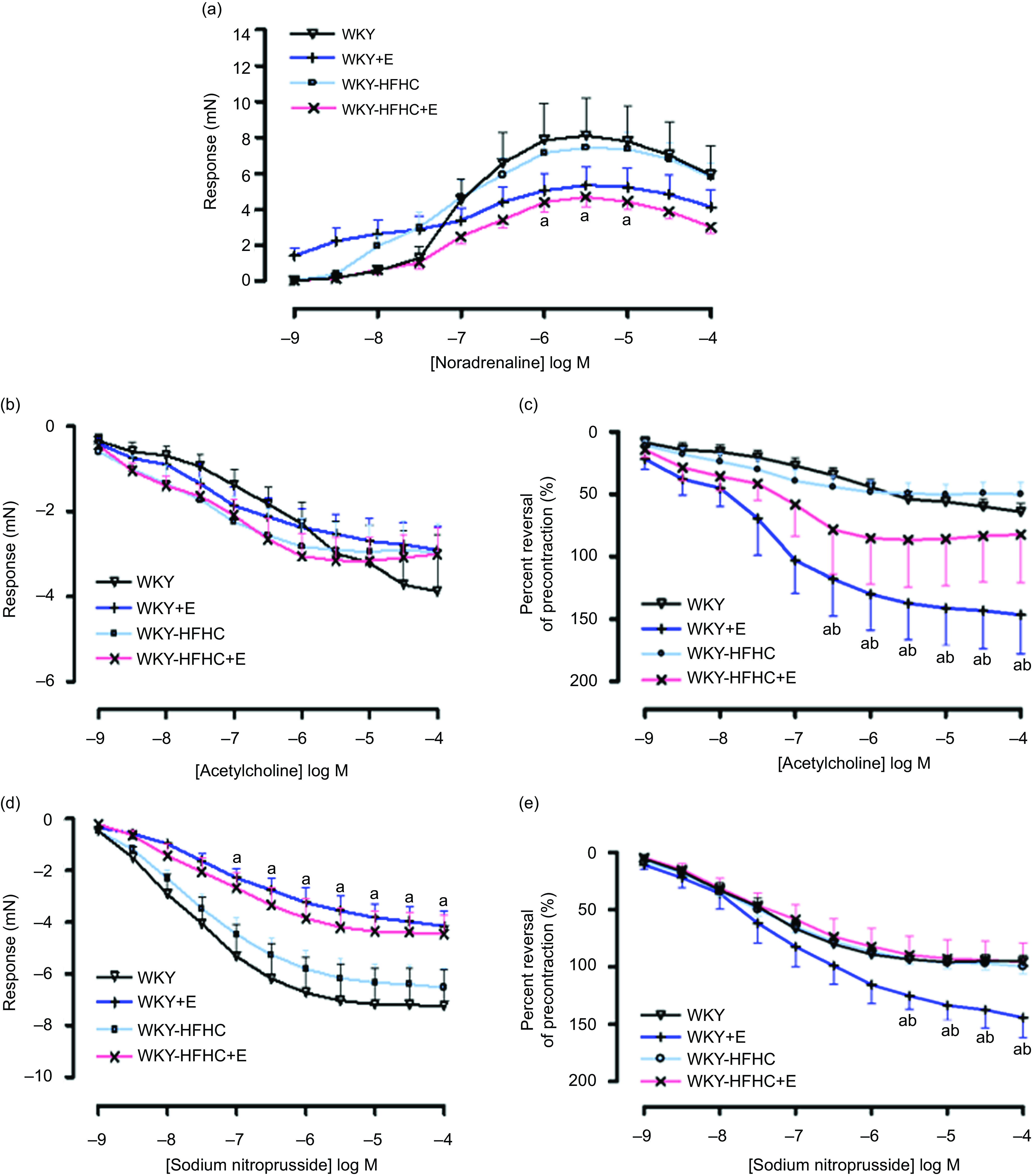
Vascular functional changes after 20 weeks of treatment. (a) Noradrenaline mediated contraction of the thoracic aorta, (b) endothelium-dependent relaxation to acetylcholine of noradrenaline pre-contracted thoracic aorta, (c) endothelium-dependent relaxation to acetylcholine of noradrenaline pre-contracted thoracic aorta expressed as percent reversal of pre-contraction (%), (d) endothelium-independent relaxation to sodium nitroprusside of noradrenaline pre-contracted thoracic aorta E. Endothelium-independent relaxation to sodium nitroprusside of noradrenaline pre-contracted thoracic aorta expressed as percent reversal of pre-contraction (%). E, epicatechin; HFHC, high-fat, high-carbohydrate. Data expressed as mean ± sem; *n* 11 for all groups ^a^ *P* < 0·05 *v*. WKY; ^b^ *P* < 0·05 *v*. WKY-HFHC (two-way ANOVA with Bonferroni’s multiple comparisons tests).

Contractile responses to noradrenaline in thoracic aortic vessels were significantly reduced in HFHC rats treated with epicatechin compared to WKY rats; however, these responses were not significantly different to untreated WKY-HFHC rats (Fig. 1(a)). There were no significant differences in endothelial-dependent relaxation responses to acetylcholine in epicatechin-treated groups (Fig. 1(b)) despite significant reductions in EC50 in all groups (Table 9). When plotted as a percent reversal of precontraction, epicatechin-treated WKY rats showed significantly increased relaxation responses to acetylcholine (Fig. 1(c)) and sodium nitroprusside (Fig. 1(e)) in thoracic aortic rings. The endothelial-independent response to sodium nitroprusside in the thoracic aorta was significantly reduced in WKY + E treated rats compared with WKY control rats (Fig. 1(d)).

**Table 9.** Log EC50 values for noradrenaline, acetylcholine and sodium nitroprusside in aortic rings of treatment and control groups

Parameters	WKY		WKY + E		WKY-HFHC		WKY-HFHC + E	
	Mean	sem	Mean	sem	Mean	sem	Mean	sem
Noradrenaline (Log EC50)	–7·031	0·10	–7·235	0·18	–7·293	0·10	–7·005	0·16
Acetylcholine (Log EC50)	–6·270	0·05	–7·078	0·06*	–7·481	0·04*	–7·435	0·08*
Sodium nitroprusside (Log EC50)	–7·560	0·04	–6·918	0·05*	–7·407	0·04*	–7·248	0·04*,†

E, epicatechin; HFHC, high-fat, high-carbohydrate. Data expressed as mean ± sem; *n* 11 for all groups.

* *P* < 0·05 *v*. WKY.

† *P* < 0·05 *v*. WKY-HFHC (two-way ANOVA with Bonferroni’s multiple comparisons tests).

### Effect of epicatechin on vascular function in mesenteric arteries of Wistar-Kyoto and Wistar-Kyoto-high-fat-high-carbohydrate rats

**Fig. 2.**
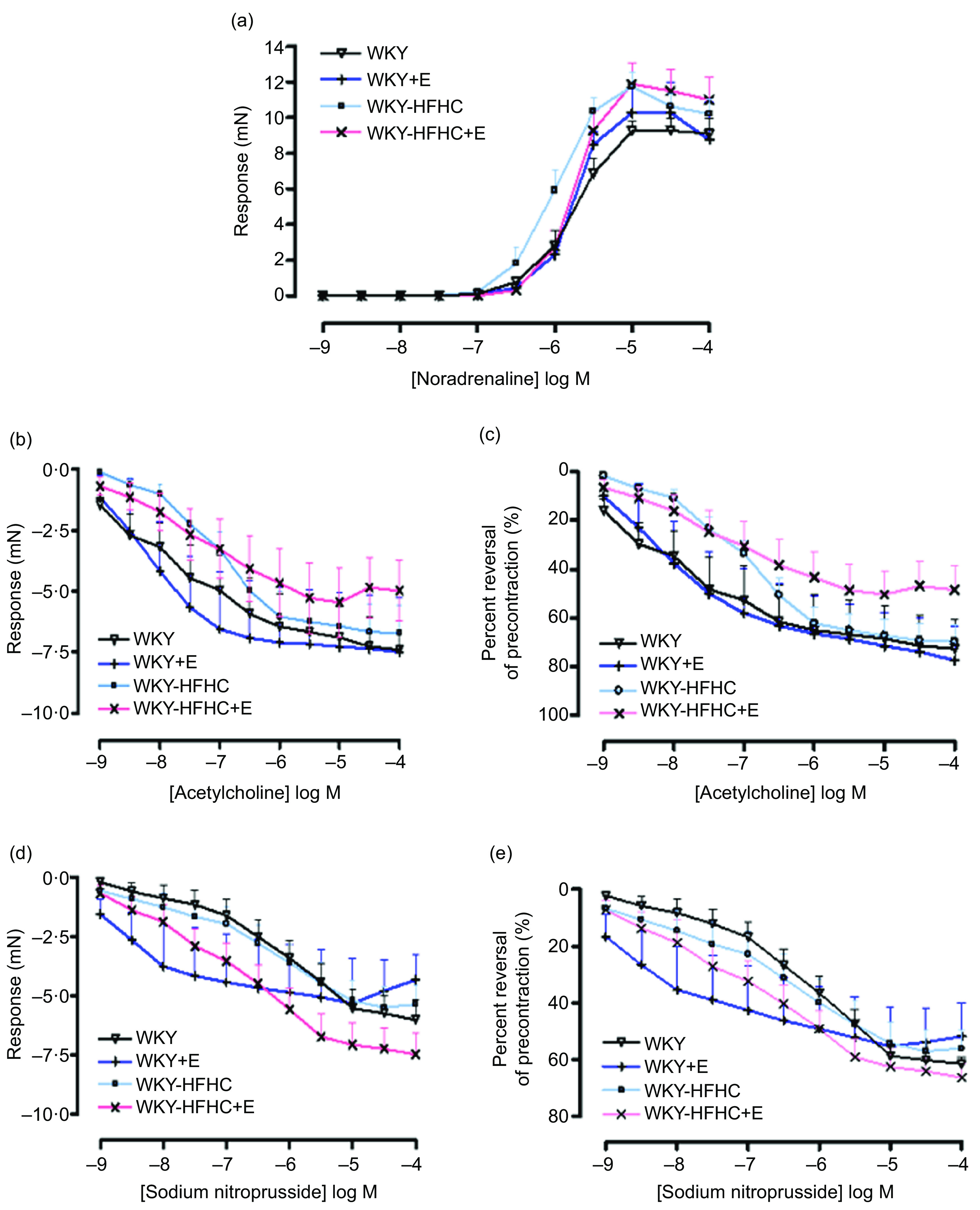
Vascular functional changes after 20 weeks of treatment. (a) Noradrenaline mediated contraction of the mesenteric artery, (b) endothelium-dependent relaxation to acetylcholine of noradrenaline pre-contracted mesenteric artery, (c) endothelium-dependent relaxation to acetylcholine of noradrenaline pre-contracted mesenteric artery expressed as percent reversal of pre-contraction (%), (d) endothelium-independent relaxation to sodium nitroprusside of noradrenaline pre-contracted mesenteric artery, (e) endothelium-independent relaxation to sodium nitroprusside of noradrenaline pre-contracted mesenteric artery expressed as percent reversal of pre-contraction (%). E, epicatechin; HFHC, high-fat, high-carbohydrate. Data expressed as mean ± sem; *n* 11 for all groups. ^a^ *P* < 0·05 *v*. WKY; ^b^ *P* < 0·05 *v*. WKY-HFHC (two-way ANOVA with Bonferroni’s multiple comparisons tests).

Epicatechin was not able to influence the contractile response to noradrenaline in mesenteric vessels in any group (Fig. 2(a)), although epicatechin-treated WKY-HFHC rats did display increased EC50 values (Table 10). There were no significant differences in either endothelial-dependent relaxation responses to acetylcholine (Fig. 2(b)) or endothelial-independent relaxation responses to sodium nitroprusside in mesenteric vessels in any group (Fig. 2(d)). Mesenteric artery relaxation responses to both acetylcholine (Fig. 2(c)) and sodium nitroprusside (Fig. 2(e)) were not significantly different in any group when plotted as percent reversal of precontraction.

**Table 10.** Log EC50 values for noradrenaline, acetylcholine and sodium nitroprusside in mesenteric arteries of treatment and control groups

Parameters	WKY		WKY + E		WKY-HFHC		WKY-HFHC + E	
	Mean	sem	Mean	sem	Mean	sem	Mean	sem
Noradrenaline (Log EC50)	–5·788	0·02	–5·781	0·06	–6·015	0·07*	–5·759	0·05†
Acetylcholine (Log EC50)	–7·318	0·18	–7·888	0·05*	–7·030	0·03	–7·143	0·08
Sodium nitroprusside (Log EC50)	–6·242	0·05	–7·897	0·19*	–6·260	0·04	–6·798	0·05*,†

E, epicatechin; HFHC, high-fat, high-carbohydrate. Data expressed as mean ± sem; *n* 11 for all groups.

* *P* < 0·05 *v*. WKY.

† *P* < 0·05 *v*. WKY-HFHC (two-way ANOVA with Bonferroni’s multiple comparisons tests).

### Effect of epicatechin on cardiac electrophysiology in Wistar-Kyoto and Wistar-Kyoto-high-fat-high-carbohydrate rats

There was no significant difference in action potential duration at 20 %, 50 % or 90 % of repolarisation in epicatechin-treated groups compared with their respective controls (Table 11). Action potential amplitude remained unchanged by epicatechin treatment in all groups (Table 11). The force of contraction of the left ventricular papillary muscles was significantly increased in both epicatechin-treated WKY and WKY-HFHC (Table 11).

**Table 11.** Cardiac electrophysiological parameters of treatment and control groups

Parameters	WKY		WKY + E		WKY-HFHC		WKY-HFHC + E	
	Mean	sem	Mean	sem	Mean	sem	Mean	sem
Action potential duration at 20 % of repolarisation (ms)	14·33	1·41	13·50	0·82	13·63	0·90	13·67	0·68
Action potential duration at 50 % of repolarisation (ms)	23·60	3·21	22·13	1·71	21·37	1·81	21·80	1·22
Action potential duration at 90 % of repolarisation (ms)	70·07	8·17	74·23	8·05	85·19	9·41	73·42	8·28
Resting membrane potential (mV)	–68·01	2·80	–66·50	2·55	–62·54	3·59	–62·86	2·38
Action potential amplitude (mV)	65·36	3·89	61·07	2·26	56·00	4·58	60·08	2·37
Force of contraction (mN)	1·35	0·23	2·66	0·40*	1·15	0·21	2·19	0·48†

E = Epicatechin. Data expressed as mean ± sem; *n* 11 for all groups.

* *P* < 0·05 *v*. WKY.

† *P* < 0·05 *v*. WKY-HFHC (two-way ANOVA with Bonferroni’s multiple comparisons tests).

## Discussion

This study aimed to investigate the potential cardioprotective effects of epicatechin, a flavanol known for its antioxidant, anti-inflammatory and anti-hypertensive properties^(17)^, in rat models of metabolic syndrome by assessing the effects of a 12-week treatment of epicatechin at a dose of 5 mg/kg/d on cardiometabolic parameters associated with hypertension and obesity in middle-aged, diet-induced rat models of metabolic syndrome. Metabolic syndrome was induced in WKY rats through HFHC feeding, which was confirmed by the presence of glucose intolerance, dyslipidaemia, abdominal obesity and hypertension. While HFHC-fed rats continued to gain weight throughout the treatment period, epicatechin treatment did not affect their food, energy or fructose intake, nor did it have a significant impact on their weight gain. This finding would indicate that epicatechin at a dose of 5 mg/kg/d may not counteract the weight gain induced by HFHC feeding.

The oral glucose tolerance test results of the current study indicated that the administration of epicatechin did not have a significant effect on glucose intolerance in WKY rats. Although previous studies have demonstrated improved oral glucose tolerance after epicatechin treatment, these positive effects were observed in diabetic Wistar^(18,19)^ and Zucker^(20,21)^ rats. Similarly, beneficial effects were also observed in Wistar rats fed a high-fat and high-fructose diets^(9,22)^. Notably, in these investigations, the improvement in glucose metabolism corresponded with a reduction in body weight, implying a potential relationship between the two factors. This lack of weight reduction in our study might have contributed to the absence of significant improvement in glucose intolerance.

Currently, it is not known whether epicatechin would be able to prevent the development of hypertension in normotensive obese animals. Consequently, epicatechin at a dose of 5 mg/kg/d was ineffective in preventing the development of hypertension in WKY-HFHC rats in the current study. Although epicatechin-treated rats in the obese group had significantly increased serum nitric oxide concentrations, the elevated levels were not sufficiently increased to influence functional blood pressure changes. This is in contrast to other rat models of hypertension studies, where epicatechin has been found to reduce elevated blood pressure in the SHR at a much higher dose of 100 mg/kg/d^(23)^ and in deoxycorticosterone acetate-salt hypertensive Wistar rats at a lower dose of 1 mg/kg/d^(24)^. At a similar dose to the current study (3 mg/kg/d), Galleano et al. found that epicatechin increased both nitric oxide levels and decreased blood pressure in SHR rats^(25)^.

Epicatechin in the current study was able to significantly reduce markers associated with oxidative stress and inflammation in obese animals. Epicatechin significantly reduced serum levels of IL-6 and 8-isoprostane in WKY-HFHC rats. In agreement with current knowledge^(26,27)^, these results indicate that epicatechin at 5 mg/kg/d is effective in reducing both inflammation and oxidative stress in states of obesity and metabolic distress.

In agreement with the literature^(28)^, epicatechin treatment significantly decreased serum TAG levels by over half in WKY-HFHC rats, while LDL concentrations were decreased by 45·9 % in WKY-HFHC rats. Serum HDL levels were increased by 27·9 % in WKY-HFHC rats. It is postulated that the mechanism behind epicatechin’s influence on plasma lipid concentrations lies in its ability to improve oxidative metabolism, thereby enhancing mitochondrial structure and function^(28)^.

A widely accepted view among researchers is that the principal mechanism through which epicatechin induces vasorelaxation involves elevating the expression and activity of endothelial nitric oxide synthase, which in turn triggers the release of nitric oxide and promotes a healthier endothelial function^(29,30)^. One study demonstrating the effect of epicatechin on mesenteric arteries from Sprague Dawley rats found that pre-treatment of vessels with epicatechin (10 µmol/l) significantly augmented the endothelial-dependent relaxation response to acetylcholine, while incubation with L-N^G^-Nitro arginine methyl ester showed a reversed response^(31)^. Similar results have been found in chronic studies^(32)^. Treatment with epicatechin at 250 mg/kg/d administered to SHR rats for 6 d significantly reduced systolic blood pressure and increased nitric oxide synthase activity in blood vessels, evidenced through enhanced aortic vessel relaxation to acetylcholine^(25)^. This is partly in agreement with data obtained from the current study. Epicatechin-treated WKY rats displayed increased relaxation responses to both acetylcholine and sodium nitroprusside in aortic rings despite no significant increases in serum nitric oxide levels observed in these rats. Although epicatechin treatment led to a significant increase in nitric oxide levels in obese rats, it remains unclear why this increase did not translate to improved endothelial function in the WKY-HFHC rats. While the literature presents conflicting evidence, it is evident that there are numerous elements involved in epicatechin’s vasoactive mechanisms. Nevertheless, it is plausible that enhanced nitric oxide bioavailability and decreased oxidative stress play pivotal roles in reducing contractile responses and enhancing relaxation responses.

PICP is a biological marker of collagen deposition commonly used to determine the extent of cardiac fibrosis in humans with hypertension and heart failure^(33)^. In WKY-HFHC rats treated with epicatechin, the serum levels of PICP were normalised, indicating that the compound may have the potential to avert the development of fibrosis triggered by HFHC feeding in rats. However, to ascertain whether the observed declines in PICP levels in these rats corresponded to diminished fibrotic alterations related to the heart, further histological examinations of the cardiac tissue would be necessary.

Although there is no existing literature reporting the impact of epicatechin on cardiac fibrosis prevention in animal models of chronic CVD, studies have demonstrated the cardioprotective effects of epicatechin in animal models of acute myocardial injury^(34,35)^. According to Sasaki et al.^(36)^, green tea catechins have an inotropic effect on cardiac tissue, which is mediated by alterations in intracellular Ca. Interestingly, the study revealed that the positive inotropic effect was only observed in cardiac tissue exposed to catechins with a galloyl moiety, and that epicatechin had no inotropic effect even at concentrations as high as 300 µM, which contrasts with the results of the current study. Epicatechin significantly augmented the contractile force in WKY + E and WKY-HFHC + E rats, implying that epicatechin might operate through other mechanisms not related to intracellular Ca modulation or that chronic epicatechin administration has a cumulative effect that may indirectly involve secondary metabolite activity. These changes may also suggest a more robust ventricular cell contractile function.

Emerging reports suggest that epicatechin might have an effect on cardiac electrical function^(6,37)^. Epicatechin significantly improved cardiac electrophysiology by reducing the resting membrane potential, action potential amplitude and force of contraction in isolated left ventricular papillary muscles of 8–10 weeks old Wistar rats^(6)^. Rats treated with apple polyphenols containing epicatechin exhibited significant reductions in action potential duration at 50 % and 90 % of repolarisation, while also preserving potassium channel density and connexin 43 expression^(37)^. Additionally, there were notable decreases in reactive oxygen species levels in these rats^(37)^. The observed reduction in oxidative stress in obese rats treated with epicatechin in the current study suggests that epicatechin’s ability to improve electrophysiological function in these animals may be partly due to its anti-oxidant and anti-fibrotic properties. By reducing the metabolism, inflammation, oxidative stress and fibrotic potential in cardiac tissue, epicatechin treatment could mitigate pathological electrophysiological changes.

The limitations of this study include the absence of insulin and HbA1c measurements in the assessment of glucose metabolism, resulting in a qualitative estimate of 24-h average blood glucose levels. Furthermore, the study’s findings were limited by the lack of histological data on the heart, adipose tissues and other organs, as well as the absence of mechanistic data supporting the observed metabolic changes. These limitations emphasise the necessity for additional research to provide a more comprehensive evaluation of the effects of epicatechin on obese rat models with metabolic syndrome.

### Conclusions

The current study investigated the potential cardioprotective effects of epicatechin in rat models of metabolic syndrome. The results showed that while epicatechin did not affect weight gain, blood pressure or glucose intolerance, it was effective in reducing markers associated with oxidative stress and inflammation, as well as improving lipid profiles. Epicatechin has also been found to enhance vascular reactivity and protect against electrical dysfunction in rat models of metabolic syndrome induced by a high-fat diet. The findings of this study provide insights into the potential therapeutic applications of epicatechin in mitigating the adverse cardiometabolic effects of metabolic syndrome, particularly in reducing markers of oxidative stress, inflammation and dyslipidaemia. Future studies investigating the effects of higher doses and longer treatment durations of epicatechin could help further elucidate the therapeutic potential of this flavanol.
